# Childhood Obesity as a Predictor of Coronary Artery Disease in Adults: A Literature Review

**DOI:** 10.7759/cureus.11473

**Published:** 2020-11-13

**Authors:** Anam Bashir, Shriya Doreswamy, Lakshmi Rekha Narra, Pinal Patel, Jesus E Guarecuco, Ayesha Baig, Simmy Lahori, Stacey E Heindl

**Affiliations:** 1 Internal Medicine, California Institute of Behavioral Neurosciences and Psychology, Fairfield, USA; 2 Otorhinolaryngology, Vydehi Institute of Medical Sciences and Research Centre, Bangalore, IND; 3 Anesthesiology, California Institute of Behavioral Neurosciences and Psychology, Fairfield, USA; 4 Neuroscience and Psychology, California Institute of Behavioral Neurosciences and Psychology, Fairfield, USA; 5 Medicine, California Institute of Behavioral Neurosciences and Psychology, Fairfield, USA; 6 Medicine, Avalon University School of Medicine, Willemstad, CUW

**Keywords:** childhood obesity, coronary artery disease, metabolic syndrome, cardiovascular disease

## Abstract

Obesity in children is becoming a worldwide epidemic that requires immediate attention. Despite all the efforts directed towards controlling this issue, its prevalence is increasing overtime both in developed and developing countries. With an increasing prevalence in the younger age groups, it is emerging as a public health crisis. A rise in body mass index (BMI) results in an increased risk of developing a variety of metabolic and cardiovascular diseases, particularly coronary artery disease (CAD). The early onset of the disease affects the peak productivity years in young individuals leading to disability at a later age. It makes it essential that we understand the contributory factors towards the development of obesity as a risk factor for CAD and develop strategies that hinder the progression towards adverse outcomes. There is an urgent need to screen these children at a younger age and educate them to change their lifestyle to decrease the BMI within the normal range to promote cardiovascular health. It requires a multidisciplinary approach involving dietary, physical, and behavioral-centered strategies. Failure to control this epidemic timely may cause widespread consequences for the quality of life and longevity.

## Introduction and background

Childhood obesity is one of the main challenges public healthcare is facing in the modern era. The prevalence of obesity has doubled in the past decade, with an increasing trend in younger generations. According to recent statistics, there was a record rise in obesity of 23% in developed and 14% in developing countries [1]. The financial burden of childhood obesity may not be only on the healthcare system, but there is also an economic impact of it. The healthcare burden may be due to direct or indirect medical costs that are not only the medical bills but also the loss of productivity and disability in obese individuals.

No doubt, obesity is multifactorial and complex in origin. Various controllable factors are associated with the development of obesity that includes general lifestyle and dietary habits. Controlling these factors can prevent morbidities related to them. Clinicians and scientists stress that obesity should be treated like any other chronic disease so that it is taken more seriously by families and society [2]. Many health problems, like the development of diabetes mellitus, coronary artery disease (CAD), obstructive sleep apnea, and large and small joint osteoarthritis, when followed back, are linked to obesity [3]. It is becoming worrisome that children have started developing diseases traditionally considered adult diseases [4]. Cardiovascular diseases are the most common causes of death globally, and CAD is the most common among heart diseases. According to the National Vital Statistic report of 2017, the mortality rate for CAD is 23.4% among the US population [5].

Studies suggest that youth who are obese as teenagers, it is 90% likely they grow up to become overweight or obese at 35 years and making pediatric obesity one of the modifiable factors that, if controlled, can prevent health issues related to it in adulthood [6]. As we already know, obesity is one of the main risk factors for the development of cardiovascular diseases but have limited knowledge about the relationship between childhood obesity and cardiovascular diseases. It is also not frequently discussed that cardiovascular risk factors may be present during childhood. Defining such relationships may help in foreseeing the long-term implications and economic costs of cardiovascular risk factors in childhood.

Since obesity in children is often under-diagnosed and under-treated, training should be provided to the practitioners to identify obesity, especially in the primary care setting [7]. Data suggest that if we can identify the risk factors for the development of cardiovascular diseases at an age when a child is transitioning into adulthood, we might change the course of high-risk youth to a low-risk adult [8]. Establishing this relationship might rationalize any interventions deliberated to improve overall health, minimize the chances of development of premature cardiovascular disease, and reduce the rates of mortality related to them [9]. Controlling obesity at a younger age with appropriate interventions should become a priority at a more inclusive and broad level. The healthcare providers hold a vital role in initiating discussions, identifying the risks, and educating the children and their families that will help promote a healthier lifestyle. 

## Review

Methods

Using PUBMED and Google scholar online databases systematically, data were collected and accredited. Using MeSH words “Childhood Obesity” and “Coronary artery disease” setting the limit between the year 2000 and 2020, the search yielded 99 papers that were all in English. After applying the inclusion criteria of the study population under 18 years of age, Human species, and full-text journal articles, 67 articles were yielded. This included literature reviews, observational, interventional studies, and clinical trials. In our final paper, we were able to use 34 articles.

Results

Using our inclusion criteria of human subjects under 18 years of age with obesity and filtering the literature that was articles in the English language only, written in the past 20 years, we were able to include only 34 in our paper. We were able to find statistics that show a rise in childhood obesity in recent times and the direct and indirect financial burden from it. The literature helped define an association between obesity and CAD with signs evident at a young age. We were also able to find literature that supports that reducing bodyweight improves cardiovascular health and the interventions that aid in lowering this risk. Table 2 summarizes the clinical trials done on children and adolescents that reveal the impact of interventions on lowering the body mass index (BMI) and that on cardiovascular health with interventions.

Discussion

Over the past few decades, obesity has become widely prevalent, both in developed and developing countries and, exhausts a great deal of healthcare budget annually. It is just not a problem for developed countries anymore. Developing countries that still are combating undernutrition now are facing an obesity pandemic. The disease burden will continue to rise, so will be the morbidities related to it if not controlled in time. Although heredity influences weight, with modernization, increased calorie intake, and decreasing levels of physical activity, an increasing trend of higher body weight is now seen more frequently in younger age groups.

Childhood obesity

According to World Health Organization (WHO), obesity is two standard deviations above the body mass index (BMI) that is normal for that age and sex, using the WHO standard growth chart as a reference for children and adolescents. The Center for Disease Control classifies obesity according to the percentile the child falls in, appropriate for that age, and a percentile equal or more than 95 classified as obesity (Table 1).

**Table 1:** Classification of obesity.

Weight status	Percentile in the growth chart
Underweight	Less than the 5th percentile
Normal or Healthy Weight	5th to 84th percentile
Overweight	85th to less than the 95th percentile
Obese	95th percentile or greater

Childhood obesity is associated with adult obesity-related morbidities that include diabetes, hypertension, dyslipidemia, and cardiovascular diseases. Obesity has been reclassified by the American Heart Association (AHA) as a ‘major, modifiable risk factor’ for CAD. If we can detect atherosclerosis at a younger age, we may prevent the progression of severe clinical events like myocardial infarction [10]. Generally, when we need to establish a correlation between a predictor of disease and morbidity, there must be a substantial relationship before we start using it as a predictive tool [11]. The goal is to find such evidence that can help us link childhood obesity with CAD and prevent it at earlier stages.

Cardiovascular risk

Cardiovascular diseases now exhaust 17% of the American budget annually, which makes health expenditures, corresponding to the BMIs, one of the highest in the world. In the past few years, the cost of cardiovascular diseases has risen drastically to an average of 6% rise annually, accounting for an upsurge in the total medical expenditures by 15%. In the next 20 years, a hike of 10% in the prevalence of cardiovascular diseases expected, the attributable cost to triple [12]. On average, for a 10-year-old child with obesity, the estimated direct medical expense in the United States, compared with a similar child with a healthy weight, is between US$12,660 and US$19,630 [13].

Although the mortality rate of CAD is decreasing, the prevalence is increasing with the rise in the frequency of risk factors, particularly obesity and diabetes [14]. When analyzed independently, data propose that obesity may be an independent risk factor for the development of atherosclerosis. The arterial abnormalities associated with atherosclerosis may manifest in obese children without developing any clinical signs and symptoms. The results from the Mendelian randomization trial done by Geng et al. show that if BMI (kg/m2) increases by one standard deviation from the mean, the risk of CAD in adult life increases by 28% [15].

Dyslipidemia

With an increase in BMI and waist circumference (WC), observed is a rise in triglyceride (TG) and a decreased level of high-density lipoprotein (HDL) that may be associated with an unfavorable outcome. Atherosclerosis is known to develop from high serum lipids and accumulation of lipids in the arterial wall. A study done on children of an average age of 9.5±1.7 years with BMI ≥95th percentile revealed a higher level of total cholesterol, TGs, and low-density lipoprotein (LDL) in the children, compared to the group of the same age and gender with waist circumference less than or equal to the 90th percentile. A study conducted on elementary school students with central obesity alongside general obesity by Bijari et al. revealed higher cholesterol, TGs, and LDL; and a lower level of HDL; and the highest number of dyslipidemias in obese children. The students who had central obesity but were not generally obese were more likely to develop a risk of hypertriglyceridemia and low HDL, compared to a control group [16].

Metabolic Syndrome

Metabolic syndrome is a combination of increased adiposity, insulin resistance, increased blood pressure, and dyslipidemia. Its prevalence is rising tremendously in the younger population leading to complications like polycystic ovarian syndrome, diabetes, and cardiovascular diseases. It is observed that WC was a common factor that linked dyslipidemia and metabolic syndrome [17]. The United States has become one of the countries with the most incidence of metabolic syndrome in adults and incidence is increasing in the younger population. Although the components that define metabolic syndrome may be present, clinicians do not often make a diagnosis of this syndrome in the pediatric population. Since it is one of the components contributing towards dyslipidemia, hence identifying and treating it may help prevent progression towards cardiovascular implications. 

Arterial Abnormalities

Atherosclerosis has a complex mechanism, and the earliest known age at which it develops is childhood. Arterial endothelial dysfunction and increased carotid intima thickness both are early markers of atherosclerosis. Atherosclerosis, which often starts as endothelial damage, proceeds to plaque formation. A study was done by Zhu et al., on Chinese obese children between ages 7 and 15 years, regarding the association between obesity and early atherosclerosis. It studied the morphological and functional changes in the arterial walls associated with obesity. The arterial wall thickening, when measured using ultrasonography in obese children compared to the lean control group, suggested that these vascular abnormalities observed may be there as early as childhood [18].

Woo et al. studied a cohort of obese asymptomatic Chinese children of ages between 9 and 12 years and observed 50% of them developed fatty streaks, one of the phases of development of atherosclerosis. It also suggested that individuals who had higher BMI may have obesity as the only recognizable arterial risk factor for the development of atherosclerosis. Other risk factors for the development of atherosclerosis like family history, smoking, etc. may have an additive effect [19]. A Mendelian randomization analysis by Geng et al. analyzes the association between higher childhood BMI and chronic diseases like diabetes, chronic kidney diseases, and cardiovascular diseases. By using data from various databases, this study shows a causal effect of obesity on CAD with a 28% increased risk in adult life [15]. A positive statistically significant association between childhood BMI and CAD in children aged 12 years and older is observed, with an increase of 11% in the risk of CAD with every kg/m2 increase in BMI [20].

The BMI predicts angiography-proven CAD in young adults who were obese. That makes the risk of coronary heart disease in adulthood, three times more common among the adolescent who has a BMI in the 86th percentile or higher. This analysis shows that a rise in BMI in adolescence proportionally raises the risk of CAD in adulthood, with adolescent at BMI in the higher decile having an incidence nearly seven times as high as that for the ones in the lowest decile [21]. Tounian et al. conducted a study comparing the arterial mechanics and endothelial function using ultrasound in 48 obese children and 27 controls. Arterial elasticity, one of the measures to check the integrity of the arterial structure and endothelial dysfunction, was observed to be compromised. An impairment in arterial-distensibility was noted in severely obese teenagers when compared to lean controls [22].

A study on 30 obese children (BMI 95th percentile or high) of ages 10-16 years by Çelik et al. shows aortic pulse wave velocity (APWV) to be high in obese children, compared to a control group of lean children. It is a sign of arterial stiffness and decreased arterial compliance. This observation may suggest that obesity may cause subclinical atherosclerosis that can be detected at an early age using noninvasive methods like conventional echocardiography [23].

Prevention

**Figure 1:**
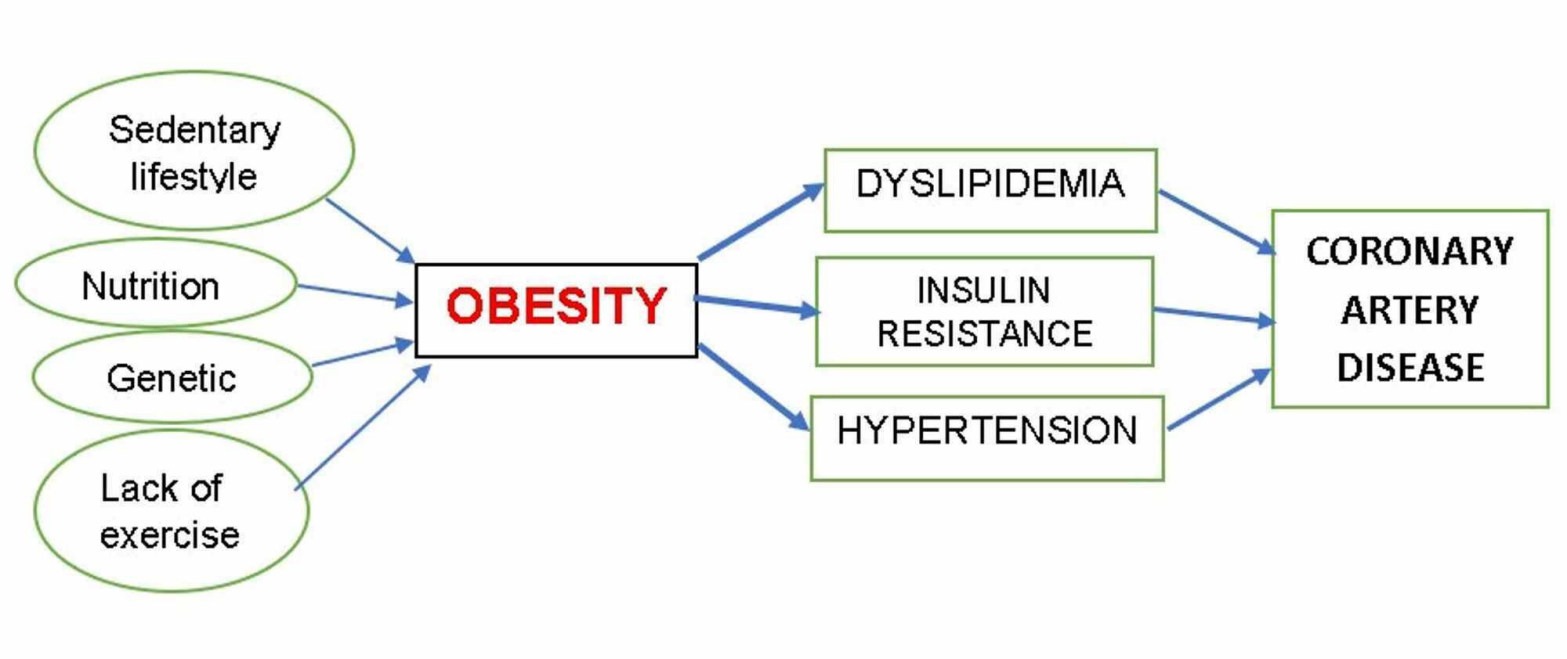
The relationship between obesity and cardiovascular diseases.

Primordial prevention is the method employed to prevent the development of risk factors, among which are the strategies that lower the risk. It involves re-education, re-orientation, and motivation of individuals in a way that either hinders the development of risk factors or reduces it to the extent that their lifetime risk drops. In this case, introducing a healthy lifestyle and eating habits is of great importance. These changes should not only be efficient but achievable that these children can adhere to and continue to follow throughout their lives, targeting to reduce the causative factors of obesity (Figure 1).

Early Screening

Every time a child visits a pediatrician, the length and weight recorded on a standard metric graph is the part of the obesity screening practice. This graph is maintained over time and tracked, which helps control rapid weight gain in children. Recommendations are to begin a routine BMI screening for overweight/obesity as early as two years of age. Similarly, recommended is the screening of all children with obesity, for hypertension, dyslipidemia, and diabetes. The healthcare professional should discuss this issue with the child and their family and offer them support.

The abnormal accumulation of lipids in the vascular wall associated with obesity is reversible, and an early screening at an early stage of atherosclerosis is an ideal opportunity for preventive measures. Early identification and intervention may reduce clinical presentation and improve long-term health outcomes [24].

Lifestyle Changes

Lifestyle modification is often stressed as the most pivotal when cardiometabolic risk is suspected and, weight loss is one of the most vital parts of it. Most individuals who are obese as children often grow up to become obese adults, which may be because childhood obesity and adult obesity have the same genetic predisposition.

Healthy dietary changes with increased physical activity with intensity starting from 20 min and stepwise increase to an hour of physical activity daily may prove to be beneficial. Furthermore, behavioral intervention programs have shown effectiveness in youth if started at younger age groups, before the teenage years [25]. A high fiber diet with an increased intake of fruits, vegetables, and a limited intake of sugars, processed food, and fat can help maintain a healthy weight. Encouraging children and educating their parents to consume a diet with a healthy balance of all food groups may be beneficial. 

Increasing Physical Activity

Clinical trials with obese children enrolled in aerobic exercises have shown improvements in arterial reactivity, without changes in overall adiposity but with a decrease in fat distribution toward visceral or abdominal fat. Clinical trials with children enrolled in an exercise program revealed an improvement in cardiovascular risk factors and atherosclerosis markers with weight loss [26]. Eliakim et al. who assessed the effect of exercise, nutritional, and behavioral intervention in obese children (BMI above 95th percentile) enrolled in a three-month intervention, showed a statistically significant reduction in BMI when compared to a control group of children. Of the intervention group, some children were enrolled for another three months to continue the same interventions and showed even significant weight loss [27]. It suggests that an active lifestyle which involves daily moderate-intensity exercise is more effective in reducing weight than short-term programs.

A randomized crossover study by Watts et al. with obese children enrolled in eight-week exercise training suggests that exercise reduces central obesity and shows an improvement in vascular dysfunction associated with obesity as the endothelial function has a critical role in the prevention of atherosclerosis [28]. Watts et al. who did a study on obese children enrolled in an eight-week exercise program compared to a control group of seven lean children, revealed improved flow-mediated vessel dilation on exertion. This vessel dilatation noted while exercising is due to the vasodilatory effect of nitric oxide released from blood vessels, and this effect sustained for four to six weeks. It suggests the need to develop an exercise program that encourages a sustained and long-term exercise routine [29].

Meyer et al. did a study on obese children between the ages of 11 and 16 years, randomized into control and intervention groups, assigned to six months of exercise or nonexercise control regimen without any calorie restriction. They recorded endothelium-dependent vasodilation and an improvement in intima-media thickness in these obese children after eight weeks of exercise training [30].

Implementing school and community-based strategies, improvement in diet and food quality, social support, and other incentives prove to be the cornerstone of the interventions for weight loss [31]. The prevention and management of cardiovascular risk factors associated with obesity depend a great deal on developing strategies that ensure the engagement of children in an active lifestyle as a physical activity seems to be a protective factor [32]. Therefore, patient education, behavioral modification, and effective counseling are essential techniques that can be beneficial for primordial prevention and eventually prevent disease development.

Multidisciplinary Approach

Multidisciplinary programs are considered the gold standard to treat obesity. To make diet and exercise routines successful, we need an environment that facilitates a healthy lifestyle. Modifying the shared family environment and family-based behavioral interventions were first developed to provide guidance and support child behavior changes towards a better lifestyle [33]. Davis et al. conducted a randomized controlled trial on Latino obese children and assigned them into three separate groups. A modified nutrition control to the first group, the second group with nutrition control plus strength training, and the third group nutrition control with strength training and aerobic exercises for 16 weeks each. A decrease in BMI was measured and compared for each group with the conclusion that the combination of diet control, strength training, and aerobic exercises was more effective than either separately [34].

There is a need for the parents to be mindful of their child's lifestyle, recognize rapidly increasing weight, and take necessary measures required to improve their health. It is of great importance to call for action to all the communities, healthcare organizations, schools, and social groups to take measures necessary to implement effective programs and policies that address appropriate nutrition, mobility, and physical activity. Steps to combat the obesity epidemic must be made a priority with these issues addressed both at the local and global levels. Governments, civil society, corporations, and other stakeholders must collaborate to bring long-lasting changes necessary for a healthy community. Table 2 summarizes the results of various dietary, exercise, and behavioral interventional studies done on obese children.

**Table 2:** Effect of interventions on BMI and cardiovascular health. RCT, randomized controlled trial; BMI, body mass index

Author	Study	Study population	Intervention	Result
Eliakim et al. [27]	Non-RCT	N(3-month intervention)=177, N(6-month intervention)=67, N(control)=25	3-month exercise, nutritional and behavioral education program in obese children, and another 3-months of the same intervention on some	A combination of exercise, nutritional, and behavioral intervention helped reduce BMI. The effect is more pronounced with a 6-month intervention
Watts et al. [28]	RCT	N(intervention)=14, N(control)=7	8-weeks of exercise training	Flow-mediated dilation impaired in obese children improved with exercise
Watts et al. [29]	RCT	N(intervention)=19, N(control)=20	8-weeks of circuit exercise training without dietary changes	Flow-mediated dilation impaired in obese as compared to controls, improved with exercising
Meyer et al. [30]	RCT	N(intervention)=33, N(control)=34	6-months of exercise program without a change in the diet plan	Reduction in BMI and intima-media thickness with improvement in flow-mediated dilation noted in the intervention group
Farpour-Lambert et al. [33]	RCT	N(intervention)=52, N(control)=22	6-months of exercise, behavioral, and exercise intervention groups with low and high intensity compared to controls	Exercise, dietary education, and counseling reduce BMI, improved flow-mediated dilation
Davis et al. [34]	RCT in Latinos	N(nutrition)=10, N(nutrition+ strength training)=9, N(nutrition+ strength+aerobic training)=15	16-weeks of nutrition and strength training separately, a combination of both and a third group that received nutrition+ strength and aerobic training	A combination of nutrition, strength training, and aerobics was the most effective in reducing BMI

Limitations

This study has limitations due to the scant number of articles available on this topic. Very few studies on the association between obesity and cardiovascular diseases that focus exclusively on the pediatric population are available. There is also limited literature on studies done on interventional measures done on reversing obesity or reducing cardiovascular risk factors. 

 The data available focus on the western demographic, and only a few studies for other populations have been done, which may affect the generalizability of the results.

## Conclusions

Obesity has emerged as one of the most common noncommunicable diseases in modern times predicted to rise in the future, with an increasing trend in pediatric populations across the globe. Childhood obesity not only has a psychological impact on the child but is one of the factors directly related to the development of coronary artery disease along with other factors like smoking, family history, diabetes, etc. Continual risk without any intervention will result in accelerated atherosclerosis, which may manifest as premature cardiovascular disease. By bringing BMI down within normal limits with lifestyle and behavioral changes, may not only benefit the child now but also reduce the incidence of adult obesity-related diseases and have lasting health benefits.

Healthcare professionals hold a central role in identifying, preventing, and treating complications related to obesity. Screening children on every well-child for their BMI, counseling them regarding a healthy lifestyle, offering support, and encouraging parental involvement is becoming increasingly important. The healthcare system needs to equip the physician to treat obesity in collaboration with a nutritionist, psychologist, and physical therapist to provide appropriate care. There is a need for an environment that encourages healthy food choices, increased physical activities, and offers assistance. There is an urgent need to create awareness and strategies promoting a healthy lifestyle from a young age that involves families, schools, and policymakers. There should be further research on methods that engage the younger population in physical activities and lifestyle that has long-term efficacy.
